# Diagnostic performance of contrast-enhanced ultrasound and enhanced magnetic resonance for breast nodules

**DOI:** 10.7555/JBR.32.20180015

**Published:** 2018-05-06

**Authors:** Cuiying Li, Haiyan Gong, Lijun Ling, Liwen Du, Tong Su, Shui Wang, Jie Wang

**Affiliations:** 1. Departments of Ultrasound, Radiology, the First Affiliated Hospital of Nanjing Medical University, Nanjing, Jiangsu 210029, China; 2. General Surgery, Radiology, the First Affiliated Hospital of Nanjing Medical University, Nanjing, Jiangsu 210029, China; 3. Radiology, the First Affiliated Hospital of Nanjing Medical University, Nanjing, Jiangsu 210029, China.

**Keywords:** conventional ultrasound, contrast-enhanced ultrasound, breast lesions, BI-RADS, magnetic resonance imaging (MRI), time–intensity curve (TIC), contrast-enhanced magnetic resonance imaging (CE-MRI)

## Abstract

In the current study, we sought to evaluate the diagnostic efficacies of conventional ultrasound (US), contrast-enhanced US (CEUS), combined US and CEUS and magnetic resonance imaging (MRI) in detecting focal solid breast lesions. Totally 117 patients with 120 BI-RADS category 4A-5 breast lesions were evaluated by conventional US and CEUS, and MRI, respectively. SonoVue was used as contrast agent in CEUS and injected as an intravenous bolus; nodule scan was performed 4 minutes after bolus injection. A specific sonographic quantification software was used to obtain color-coded maps of perfusion parameters for the investigated lesion, namely the time-intensity curve. The pattern of contrast enhancement and related indexes regarding the time-intensity curve were used to describe the lesions, comparatively with pathological results. Histopathologic examination revealed 46 benign and 74 malignant lesions. Sensitivity, specificity, and accuracy of US in detecting malignant breast lesions were 90.14%, 95.92%, and 92.52%, respectively. Meanwhile, CE-MRI showed sensitivity, specificity, and accuracy of 88.73%, 95.92%, and 91.67%, respectively. The area under the ROC curve for combined US and CEUS in discriminating benign from malignant breast lesions was 0.936, while that of MRI was 0.923, with no significant difference between them, as well as among groups. The time-intensity curve of malignant hypervascular fibroadenoma and papillary lesions mostly showed a fast-in/fast-out pattern, with no good correlation between them (kappa**<**0.20). In conclusion, the combined use of conventional US and CEUS displays good agreement with MRI in differentiating benign from malignant breast lesions.

## Introduction

Breast magnetic resonance imaging (breast MRI) is a well-consolidated diagnostic technique, with precise indications in the breast cancer field. Both contrast-enhanced ultrasound (CEUS) and magnetic resonance imaging (MRI) of the breast are based on the blood supply of breast nodules, and reflect enhanced morphology and hemodynamic characteristics of the nodules. These two methods have common features. The diagnostic efficiencies and time-intensity curves (TICs) of these two methods have been reported in previous studies, which included relatively small sample sizes and yielded discrepant conclusions. In addition, breast tumors were not grouped by size, and the pathological type was not taken into consideration. Therefore, this study aimed to further compare these two methods in patients with BI-RADS category 4A-5 breast lesions.

## Patients and methods

### Patients

A total of 117 patients with BI-RADS category 4A-5 breast lesions detected by conventional ultrasound (US) in the Breast Surgery Department of First Affiliated Hospital, from March 2011 to September 2016, were enrolled in this study. All patients gave their informed consent for the study and approval of the institutional review boards was obtained for the study protocol. The inclusion criteria were the following: 1) patients who had an untreated breast mass (lesion of interest) that was revealed by unenhanced ultrasound, 2) patients who were scheduled to undergo pathologic examination, and were 20 years old or older at the time of giving consent. The exclusion criteria were the following: drug allergy, arteriovenous shunt (right-left) in the heart or lungs, severe heart disease, or severe lung disease; pregnancy, suspicion of pregnancy, or active breast-feeding; any contraindications for contrast-enhanced MRI. There were 120 breast lesions in all, including three cases of bilateral lesions. All patients were female and aged from 22–76 years (median, 43 years; age), with lump diameters ranging between 4 and 40 mm [(11.18± 13.42) mm]. Conventional ultrasound (US), contrast-enhanced ultrasound (CEUS), and enhanced MRI were performed. All pathological samples were obtained by surgery or core-needle biopsy.

### Ultrasound

A Mylab Twice color Doppler was used with linear and contrast probe frequencies of 4–13 MHz and 3–9 MHz, respectively. The contrast agent was SonoVue (Bracco, Italy), mixed with 5 mL saline and vortexed to prepare a suspension of SF_6_ microbubbles.

2D ultrasound images were obtained by conventional ultrasound, and size, position, profile, boundary, and internal echo for all tumors were recorded in detail. The probe was pressurized and morphological changes of the lesions were observed. Blood flow signal display and distribution were observed by a color Doppler. When blood flow was displayed, maximum systolic velocity (Vmax), resistance index (RI) and other parameters were measured. After identifying the most abundant section of blood flow, the probe was changed as contrast probe, with 2.4 mL of SonoVue injected intravenously at the elbow vein. The contrast enhancement pattern of the lesion, and the migration and distribution of microbubbles in the focus were observed dynamically for 4 minutes. Then, regions of interest (ROIs) were selected within the breast tumor and the surrounding normal mammary gland tissue, and TICs were analyzed. Entry time for lesions and normal tissues was recorded, as well as time to peak (TP), time to achieve the peak (TTP=peak time- developing time), peak intensity, sharpness, and area under the curve (AUC).

### MRI

The contrast agent for MRI was Gd-DTPAMRI, and MRI was performed on a Siemens Trio Tim 3.0 T superconducting magnetic resonance scanner with a breast specific 8 channel phased array coil. The patient was placed on the coil by the head-first method with two breasts falling naturally. T2-Tirm scan, T1WI dynamic enhanced scan, and selective T2WI delayed sagittal scan were performed for both breasts.

For axial T2-Tirm plain scan, TR/TE was 5,000 ms/61 ms, with a slice thickness of 4.0 mm. Dynamic enhanced scan (1+5) was performed by transverse axis T1WI, with 3D-FLASH (fast small angle excitation 3D imaging sequence) and fat suppression; TR/TE was 4.23 ms/l.57 ms, with a turning angle of 10 degrees, a matrix of 256×256, a field of view (FOV) of 340×340, and a slice thickness of 0.9 mm. Plain scan was carried out once before contrast agent injection. Next, the contrast agent (Gd-GTPA, 15 mL; flow rate of 3 mL/second) was injected by the bolus approach, followed by immediate injection of 20 mL saline. At 23 seconds, enhanced scan was started and continued without interruption for 5 rounds. The parallel acquisition method was employed with a total scan time of 6 minutes and 23 seconds. Multiple reconstruction and volume reconstruction were performed with the post-processing function of the MRI device. Finally, enhanced morphology and dynamic enhanced curves of the tumor were analyzed.

### Operating procedures

The size and internal echo of each breast lesion were observed by conventional two-dimensional ultrasound (baseline sonography). Then, in the section of the suspicious lesion, the Esaote MyLab Twice ultrasound contrast function was started (mechanical index<0.2). The contrast agent was injected into an antecubital vein according to the manufacturer’s instructions, while starting the built-in timer of the ultrasound instrument. The ultrasound contrast process was continuously saved in the original data format into the hard disk of the instrument in real-time. Next, the enhanced morphological features were analyzed, and the quantitative parameters of the lesions and normal tissues were assessed with the TIC software. Plain and enhanced MRI procedures were performed on the breast, and enhanced morphological features and dynamic enhancement curves were analyzed. Finally, pathological data were obtained by biopsy or surgical resection. Imagine analysis was performed by investigators with more than five years’ experience in CEUS or MRI.

### Statistical analysis

All data were analyzed by the SPSS 13 statistical software. Pathology or coarse needle biopsy results were considered the gold standard. Sensitivities, specificities, and areas under the ROC curves in detecting breast tumors for US, CEUS, combined US and CEUS, and MRI were determined. The consistency for examining nodules by the above techniques was evaluated by Kappa statistics. *P*<0.05 was considered statistically significant.

## Results

### Demographic and baseline characteristics of the study population

The malignant nodule group included 74 women aged 22–76 years, with a median age of 46 years. The benign nodule group included 46 cases aged 20–65 years, with a median age of 40 years. Tumor size ranged from 5 to 40 mm [mean, (23.90±16.86) mm] for the malignancy group, and 4–41 mm [mean, (12.77±7.42) mm] for benign lesions. Among the 120 breast tumor cases, 46 were benign, including 13 cases of fibroadenoma, 15 cases of hyperplasia, 3 cases of intraductal papilloma, 2 cases of sclerosing adenosis, and 2 cases of inflammatory mass cases, as well as 1 case of galactostatic cyst with calcification, 7 cases of fibrocystic breast disease combined with intraductal papilloma, and 3 cases of fibrous cystic breast disease with fibroadenoma and papilloma. There were 74 malignant tumors, including 37 cases of invasive ductal carcinoma, 18 cases of invasive ductal carcinoma with intraductal carcinoma, 12 cases of intraductal carcinoma, 1 case of invasive ductal carcinoma with mucous adenocarcinoma, 2 cases of invasive lobular carcinoma, 1 case of micro papillary carcinoma, 1 case of phyllodes tending to differentiate into liposarcoma, and 2 cases of papillary carcinoma.

### Diagnostic performance of conventional ultrasound combined with CEUS and MRI

**Fig.1:**
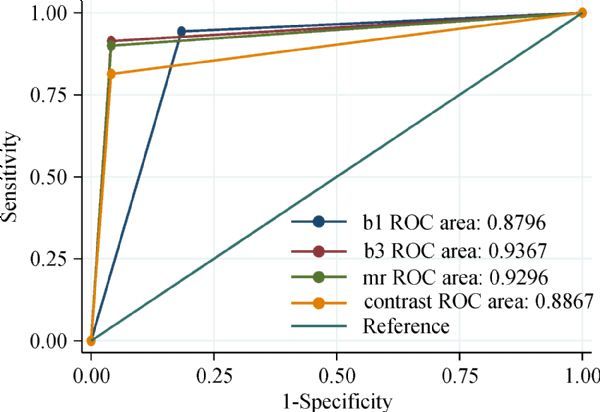
ROC curves for conventional ultrasound combined with contrast-enhanced ultrasound and enhanced MRI in evaluating breast lesions. b1: conventional ultrasound, b3: the conventional ultrasound combined with contrast enhanced ultrasound, mr: enhanced MRI.

By both conventional ultrasound combined with CEUS and enhanced MRI, 49 and 60 cases were identified as benign and malignant, respectively. The results of 11 cases were inconsistent (***Table 1***). The diagnostic performances of the two methods were assessed based on postoperative pathological results as the gold standard (***Fig. 1***). For conventional ultrasound method, the area under the ROC curve of 0.879 was obtained, with sensitivity, specificity, accuracy, positive predictive value, and negative predictive value of 92.96%, 81.63%, 88.33%, 86.1%, and 92.7%, respectively. For the conventional ultrasound combined with CEUS, the area under the ROC curve of was 0.936, and sensitivity, specificity, accuracy, positive predictive value, and negative predictive value were 90.14%, 95.92%, 92.52%, 89.9% and 100%, respectively. For enhanced MRI, the area under the ROC curve of 0.923 was obtained, with sensitivity, specificity, accuracy, positive predictive value, and negative predictive value of 88.73%, 95.92%, 91.67%, 94.6%, and 97.8%, respectively. There were significant differences between conventional ultrasound and enhanced MRI (*P*=0.008). There were no significant differences between conventional ultrasound combined with CEUS and enhanced MRI (*P*=0.251), and both had comparable diagnostic values. Examinations of breast nodules by CEUS and enhanced MRI were consistent (Kappa=0.803, *P*<0.001). Three groups were obtained according to nodule size, and areas under the ROC curves were determined for the CEUS and enhanced MRI methods (***Table 2***).

**Tab.1:** Diagnostic results of 120 breast nodule cases by conventional ultrasound combined with contrast-enhanced ultrasound and enhanced MRI (***n***)

Ultrasound/contrastenhanced ultrasound	Enhanced MRI	
	Benign	Malignant
Benign	49	5
Malignant	6	60

**Tab.2:** Areas under the ROC curves obtained by conventional ultrasound combined with contrast-enhanced ultrasound and enhanced MRI for patients with different nodule sizes

Groups	Ultrasound/contrastenhanced ultrasound	Enhanced MRI	χ^2^	*P*
<10 mm	0.907	0.952	0.46	0.495
10 mm–20 mm	0.889	0.864	0.34	0.562
>20 mm	1.000	0.976	1.00	0.317

**Fig.2:**
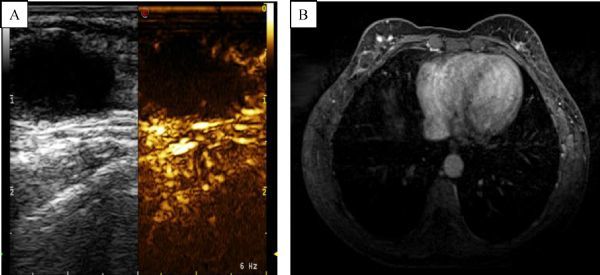
Contrast-enhanced ultrasound results in a patient with a galactocele. A: No obvious enhancement was found; B: In the same patient, enhanced MRI results also showed no significant enhancement.

**Fig.3:**
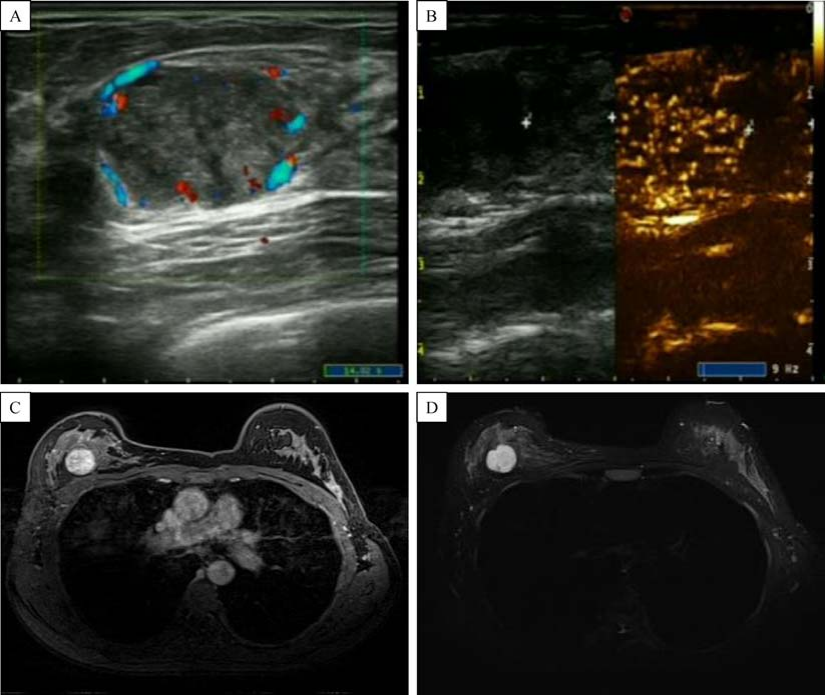
The feture of atypical fibroadenoma. A: The morphology acquired from conventional ultrasound was irregular, with nonhomogeneousinternal echo; peripheral blood flow could be seen, with internal blood flow slightly increased; B: Contrast-enhanced ultrasound was homogeneous, with high enhancement and a clear boundary; C-D: Internal enhancement was non-homogeneous, showing high enhancement. Pathology indicated a fibroadenoma.

**Fig.4:**
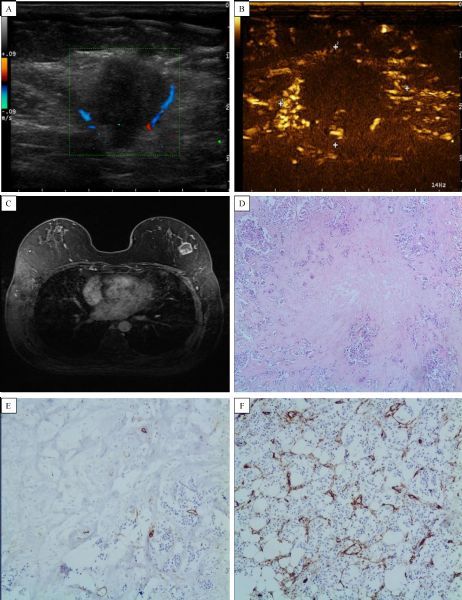
Infiltrating ductal carcinoma examination by contrast-enhanced ultrasound. A: Peripheral blood flow could be seen, and internal blood flow was not obvious, with a lesion size of about 20 mm × 16 mm. B: Peripheral radiate enhancement could be seen, with internal enhancement slightly low and uneven. C: Enhanced MRI data for the same patient showing a peripheral ring and slightly low internal enhancement. D-F: Pathology data showing internal necrosis and collagenization (CD34 indicated a small collagenization region in the tumor center); small vessels in the tumor periphery were more dense than in the tumor center region (upper figure). Sizes were consistent in contrastenhanced ultrasound, enhanced MRI, and postoperative pathology. Pathological data showed infiltrating ductal carcinoma, grade Ⅱ-Ⅲ.

**Fig.5:**
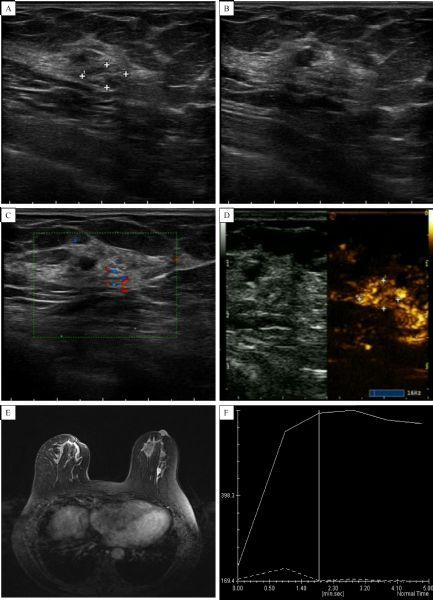
The feture of intraductal carcinoma. A-C: Conventional ultrasound showing multiple echogenic aggregation in the range of 9.5 mm × 5.0 mm, and small amounts of blood flow. D: In the same patient, after contrast-enhanced ultrasound, inhomogeneous and high enhancement was observed, with no significant enhancement surrounding the normal breast tissue. E: Enhanced MRI showing inhomogeneous and high enhancement; the curve exhibited a plateau. Pathology data showing intraductal carcinoma of grade Ⅱ-Ⅲ with two small infiltrates, and an infiltrating area of < 1 mm.

Enhanced features of breast masses are consistent between contrast enhanced US and enhanced MRI (***Fig. 2–5***).

Variance analysis was carried out for nodule size in 120 cases of breast nodules measured by CEUS and magnetic resonance imaging, and pathological methods showed no statistically significant differences. Good consistency was obtained for enhanced features of breast mass after CEUS contrast and enhanced MRI (Kappa=0.543, *P*<0.001).

***Table 3*** shows nodule size measurement by CEUS, magnetic resonance imaging, and pathological methods.

**Tab.3:** Nodule size measurement by contrast-enhanced ultrasound, magnetic resonance imaging, and pathological methods

Methods	Cases	Mean±SD (mm)	*F*	*P*
Contrast-enhanced ultrasound	120	18.60±12.72	0.00	0.99
MRI	120	18.81±14.93		
Pathology	120	18.53±11.99		

### Time-intensity curves of breast nodules by CEUS and enhanced MRI

CEUS of benign lesions showed 17 cases with fast in-fast out (36.2%), one with fast in/slow out (2.1%), 18 with slow in/slow out (38.3), and 7 with equivalent in and out (14.9) patterns; 2 cases showed no enhancement. Malignant lesions included 58 cases with fast in and fast out (78.4%), 6 with fast in and slow out (8.1%), 2 with slow in and fast out (2.7%), 7 with slow in and slow out (9.4%), and 1 with equivalent in and out (1.3%) patterns; 70, 27, and 21 cases had enhanced MRI elution, plateau, and gradual increasing types, respectively, while 2 cases showed no enhancement. In 120 cases of benign and malignant lesions, there was no significant consistency of elution curves for the fast in and fast out pattern in CEUS and enhanced MRI (malignant cases, kappa=0.048, *P*=0.284; benign cases, kappa=0.175, *P*=0.142).

## Discussion

The biological characteristics of malignant breast tumor growth and metastasis depend on tumor angiogenesis to a large extent^[1]^, and detection of tumor angiogenesis is helpful for diagnosis, efficacy evaluation, and prognostic estimation of tumors. There are several non-invasive methods for detecting angiogenesis in breast cancer, and ultrasound and MRI are widely used clinically. Conventional ultrasound can only examine large blood vessels with a diameter greater than 100–200 μm^[2]^, and is not suitable for tiny blood vessels. The average vessel diameter for the second generation ultrasound contrast agent Sonovue is only 2.5 μm, and it can be used to reveal the tumor microcirculation, explore tiny blood vessels, and perform Doppler blood flow tracing. CEUS, enhanced MRI, and Doppler ultrasound provide more tools for the evaluation of tumor vessels.

Currently, there are no uniform diagnostic criteria for distinguishing benign and malignant breast nodules by CEUS. According to reports by other investigators^[3^–^8]^, clear boundary, uniform enhancement in the whole lesion, internal vessels arranged regularly, dendritic branching blood vessels, separate strip enhancement, no enhancement, and no change were defined as benign features in this study, while peripheral radial ring enhancement, internal weaker enhancement than the peripheral one, distorted and disordered vessels, uneven enhancement of the filling defect, and range increase were defined as malignant features.

The accuracy of conventional ultrasonography in diagnosing breast nodules was 88.33%, which is consistent with that reported by Costantini *et al.* ^[9]^. The area under the ROC curve for conventional ultrasound combined with CEUS (b3) examination was 0.936, for sensitivity, specificity, and accuracy of 90.14%, 95.92%, and 92.5%, respectively; the area under the ROC curve for enhanced MRI of 0.923 was obtained, for sensitivity, specificity, and accuracy of 88.73%, 95.92%, and 91.67%, respectively, with no statistically significant differences between the two methods (*P*=0.251). The diagnostic values are consistent. Conventional ultrasound combined with CEUS with a high concordance index (Kappa=0.803, *P*<0.001) with MRI in evaluating breast nodules. In subgroup analysis based on size, similar areas under the ROC curves were obtained for CEUS and enhanced MRI, and diagnostic efficiencies were consistent, corroborating previous reports by other investigators^[8^,^10^–^11]^. In CEUS, cases of BI-RADS category 4A were reduced to BI-RADS 3 category cases, which reduced unnecessary biopsies and the psychological burden on patients, achieving the same downgrade effect as MRI. Some BI-RADS 4B lesions were classified by CEUS combined with US as class 4B, as by MRI; the pathological results were sclerosing adenosis, intraductal papillary lesions, or low-grade ductal carcinoma. Four cases of low-grade ductal carcinoma were found in this study, including 3 cases with small lesions of≤1 cm; conventional ultrasound showed low echo and punctate strong echo; in CEUS, high enhancement and micro calcification through molybdenum target was observed, while enhanced MRI displayed enhancement of the corresponding molybdenum target, with the same range as CEUS. Therefore, diagnostic efficiency is consistent between the two methods. Only in one case, the distribution range of mammography calcification was wide. In this case, no obvious punctate echo was found in conventional ultrasound with only a low echo in the range of about 6×5 mm, while CEUS showed enhancement in the low echo area compared with the surrounding normal tissue and no large area enhancement; enhanced MRI showed large area of non-mass like enhancement, showing advantages compared with conventional ultrasound and CEUS, consistent with Strobel^[12]^.

The pathological findings of radial scar and sclerosing adenosis cases sometimes could not be distinguished from breast cancer; pathological types for papillary lesions are much complex, and the degree of atypical hyperplasia should be confirmed by conventional pathology or immunohistochemistry^[13]^. Conventional ultrasound, CEUS, and enhanced MRI reflected the macro features of breast nodules, and the results for BI-RADS 4B are consistent with puncture and rapid pathological biopsy. The radial scar is formed by an elastic fiber core radially around the duct and lobular structure (expressed as epithelial hyperplasia, ductal dilatation, adenosis, multiple breast papillomatosis, and calcification were seen in epithelial hyperplasia and adenosis, leading to increased lesion hardness and affecting diagnostic accuracy for radial scar by elastography to a certain extent^[14]^. CEUS and enhanced MRI were helpful in the diagnosis of radial scar, sclerosing adenosis, and intraductal papillary lesions; however, it remained difficult to detect malignancy, in agreement with Xia *et al.* ^[15]^ and Linda *et al.* ^[16]^.

Higher resolution for the soft tissue could be achieved by MRI, which has a sensitivity reaching 90%^[17]^; however, its specificity is low (37%–97%)^[18]^. Such a difference might be related to the patients selected, patient age, pathological type of the tumor, and different evaluation standards^[19]^. In this study, groups were formed according to lesion size. For lesions with a size of less than 1 cm, consistent diagnostic efficacy was achieved by conventional ultrasound combined with CEUS and MRI. However, the prerequisite was that the lesion could be detected by conventional ultrasound. For lesions not detectable by conventional ultrasound, CEUS could not be performed. Enhanced MRI has the advantages of comprehensiveness, intuition, and high sensitivity; indeed, MRI could detect small lesions or enhancement along the extension direction of the catheter unintentionally. In addition, for microcalcification, which could not be detected by conventional ultrasound, enhanced MRI shows the corresponding enhancement of the suspected target site indicated by mammography; therefore, MRI is superior to ultrasound in examining this part of the lesion^[12]^.

The benign lesions in this study included 17 cases of fast in/fast out (36.2%), one of fast in/slow out (2.1%), 18 of slow in/slow out (38.3), and 7 of equivalent in and out (14.9) patterns, while 2 cases showed no enhancement; in malignant lesions, 58 cases of fast in/fast out (78.4%), 6 cases of fast in/slow out (8.1%), 2 cases of slow in/fast out (2.7%), 7 cases of slow in/slow out (9.4%), and 1 case of equivalent enter and out (1.3%) patterns were found; meanwhile, 70, 27, and 21 cases of enhanced MRI elution, plateau, and gradual increasing types were observed, respectively, while 2 cases displayed no enhancement. The fast in/fast out pattern was found in TICs of both fiber fibroadenoma and papilloma cases with malignant lesions and rich blood supply as examined by CEUS and enhanced MRI. However, the Kappa value was less than 0.20 for the fast in/fast out curve type for all cases; in malignant and benign cases examined by CEUS and enhanced MRI, no obvious correlation was observed, in agreement with reports by Caproni *et al.* ^[10]^ and Ricci *et al.* ^[11]^ and corroborating findings by Alamo *et al.* ^[20]^ and Reinikainen *et al.* ^[21]^ that after injection of the first generation ultrasound contrast agent, no consistency exists between the hemodynamics and time-intensity curve obtained with magnetic resonance data. Although both CEUS and enhanced MRI could reflect microcirculation reperfusion in breast tumor, ultrasound contrast agents only exist in blood vessels, not crossing the vascular endothelium. Meanwhile, MRI contrast agents diffuse into the extravascular space. In tumor angiogenesis, not only the number of blood vessels increases, there are also abnormal arteriovenous fistula and changes in vascular permeability; the MRI contrast agent (Gd-DTPA) in the extracellular interstitial space would be distributed in areas with rich blood supply, certain vascular permeability, and extracellular space in the body. Therefore, enhanced MRI could better reflect the characteristics of tumor blood vessels. Sonovue, a second generation ultrasound contrast agent with a microbubble diameter of 2–5μm, is a pure blood pool imaging agent, which only reflects the number of blood vessels. Li *et al.* suggested that the TIC in enhanced MRI characterizes the process of the contrast agent across the tumor arteries and capillaries, as well as vascular infiltration into the tissue space and eventually into veins, representing the transport rate and degree of contrast agent transport from the intravascular tissue space to the extravascular one; meanwhile, ultrasound contrast agents only exist in the intravascular space, only reflecting contrast agent perfusion in the intravascular space; pharmacokinetic differences between these two types of contrast agents caused no obvious correlation of the TICs^[22]^.

Although curve types between CEUS and enhanced MRI were different, CEUS combined with conventional ultrasound or elastography imaging achieved similar diagnostic efficacy as MRI, and would greatly improve diagnostic accuracy, reduce unnecessary biopsy and psychological burden, and shorten the inspection time. However, CEUS can only detect suspicious lesions, unlike MRI, which examines the breast comprehensively, and lacks comprehensiveness and intuition. In clinical work, comprehensive considerations should be made in selecting the optimal examination method.
